# ERK3 Is Required for Metaphase-Anaphase Transition in Mouse Oocyte Meiosis

**DOI:** 10.1371/journal.pone.0013074

**Published:** 2010-09-29

**Authors:** Sen Li, Xiang-Hong Ou, Zhen-Bo Wang, Bo Xiong, Jing-Shan Tong, Liang Wei, Mo Li, Ju Yuan, Ying-Chun Ouyang, Yi Hou, Heide Schatten, Qing-Yuan Sun

**Affiliations:** 1 State Key Laboratory of Reproductive Biology, Institute of Zoology, Chinese Academy of Sciences, Beijing, China; 2 Graduate School, Chinese Academy of Sciences, Beijing, China; 3 Department of Veterinary Pathobiology, University of Missouri, Columbia, Missouri, United States of America; Institute of Zoology, Chinese Academy of Sciences, China

## Abstract

ERK3 (extracellular signal-regulated kinase 3) is an atypical member of the mitogen-activated protein (MAP) kinase family of serine/threonine kinases. Little is known about its function in mitosis, and even less about its roles in mammalian oocyte meiosis. In the present study, we examined the localization, expression and functions of ERK3 during mouse oocyte meiotic maturation. Immunofluorescent analysis showed that ERK3 localized to the spindles from the pre-MI stage to the MII stage. ERK3 co-localized with α-tubulin on the spindle fibers and asters in oocytes after taxol treatment. Deletion of ERK3 by microinjection of ERK3 morpholino (ERK3 MO) resulted in oocyte arrest at the MI stage with severely impaired spindles and misaligned chromosomes. Most importantly, the spindle assembly checkpoint protein BubR1 could be detected on kinetochores even in oocytes cultured for 10 h. Low temperature treatment experiments indicated that ERK3 deletion disrupted kinetochore-microtubule (K-MT) attachments. Chromosome spreading experiments showed that knock-down of ERK3 prevented the segregation of homologous chromosomes. Our data suggest that ERK3 is crucial for spindle stability and required for the metaphase-anaphase transition in mouse oocyte maturation.

## Introduction

In mammalian oocytes, synapsis and meiotic recombination occur during fetal development, after which the oocyte enters prophase I arrest, or diplotene arrest. The diplotene arrest in oocytes may last for several months or years in the follicular microenvironment depending on the species[1], [2], [3]. Subsequently, the immature oocyte is encased as part of the primordial follicle, as it enters a growth stage. Once the female is sexually mature, in humans years after the oocyte entered meiosis, the oocyte completes meiosis I (MI) just before ovulation. Meiotic resumption from diplotene arrest is morphologically chracterized by geminal vesicle breakdown (GVBD). After GVBD, chromosomes regulate the assembly of spindle microtubules during prometaphase, and the spindle is then maintained in prometaphase I with chromosomes maintained in the spindle's central region[4]. Only at the end of this period, the kinetochores become activated so that microtubule fibers can stably connect to them. Subsequently, the chromosomes align at the metaphase plate and anaphase I (AI) ensues, followed by extrusion of first polar body. The meiotic cell cycle becomes arrested again at metaphase-II(MII) until fertilization.

Spindle assembly checkpoint (SAC) is referred to as a high fidelity surveillance system for somatic cells in mitosis to monitor accurate chromosome separation. The major components of the SAC pathway include Mad1, Mad2, BubR1 (Bub1-related kinase or MAD3/Bub1b), Bub1 and Bub3[5], [6]. During the prometaphase stage, all SAC proteins localize to unattached kinetochores which, at the same time, provide a platform to accelerate SAC complex formation. Unattached or improperly attached chromosomes activate the SAC pathway and induce mitotic checkpoint complex (MCC) establishment including BubR1, Mad2 and Bub3[7]. Essential to its function in spindle stability is the attachment to kinetochores, proteinaceous structures that assemble at the centromere of each sister chromatid[8], [9]. Kinetochores serve at least three functions:attaching chromosomes to the spindle, controlling chromosome movement, and maintaining SAC[6], [10], [11], [12]. Microtubules are metastable polymers of α-and β-tubulin subunits that switch between phases of growth and shrinkage, a process known as dynamic instability[13]. In mitosis, erroneous kinetochore–microtubule attachment, with either both sister kinetochores attached to the same pole (syntelic attachment), or both poles attached to the same kinetochore (merotelic attachment), can result in inaccurate segregation of sister chromatids and subsequent aneuploidy.

Extracellular signal-regulated kinase 3 (ERK3) is generally known as an atypical member of the mitogen-activated protein (MAP) kinase family. Despite nearly 50% identical to ERK1/2 in the kinase domain, ERK3 presents striking differences in structure from classical MAPKs. Most notably, ERK3 displays the Ser-Glu-Gly sequence instead of conserved Thr-Xaa-Tyr motif in the activation loop and ERK3 has a unique C-terminal extension terminal of 178 amino acids, which is demonstrated to link its stability[14], [15]. Benjamin and his colleagues reported cloning and characterization of the mouse ERK3 gene[16]. In mitosis, it is known that BRAF,which encodes a RAS-regulated kinase that mediates cell growth and malignant transformation kinase pathway activation,regulates ERK3 expression[17]. ERK3 interacts with MK5 (MAPK-activated protein kinase 5) *in vivo* and *in vitro* [18], [19]. Cdk1 and Cdc14 regulate the stability of ERK3 by controlling phosphorylation in its C-terminal domain[15]. Subsequent studies showed that ERK3-deficient mice displayed pulmonary immaturity, intrauterine growth restriction and neonatal lethality[20].

It is widely known that the MAPK signaling pathway (MOS/MEK/MAPK/p90rsk) plays a critical role in the regulation of mouse oocyte maturation[21], [22], [23], [24], while the roles of ERK3 are unclear. In our study, we employed microinjection of specific morpholino to delete ERK3 to investigate its function in meiosis. Cold treatment combined with ERK3 deletion was used to examine the interaction between kinetochores and microtubules.

Our results indicated that ERK3 deletion arrested oocyte maturation at the MI stage by disrupting the attachment between kinetochores and microtubules and activating the SAC component BubR1. The results provide evidence to show that ERK3 is required for spindle stability and metaphase-anaphase transition during mouse oocyte maturation.

## Results

### Subcellular localization and expression of ERK3 during mouse oocyte meiotic maturation

**Figure 1:**
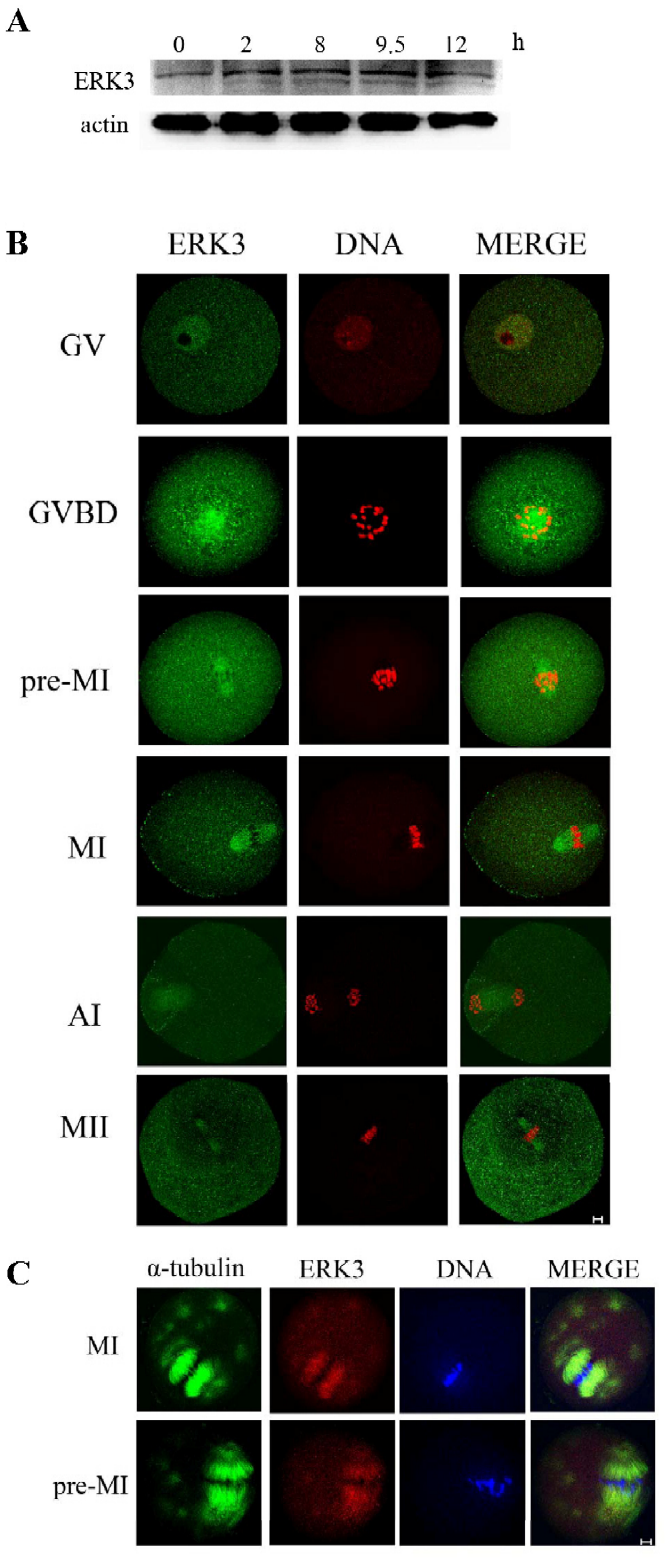
Subcellular localization, expression of ERK3 and localization of ERK3 treated with spindle-perturbing agents. (A) Samples were collected after oocytes had been cultured for 0.,2, 8, 9.5 and 12 h, corresponding to GV, GVBD, pre-MI, MI,ATI and MII stage, respectively. The molecular mass of ERK3 is 100 kDa and that of β-actin is 42 kDa. (B) Confocal microcopy showing immunostaining of ERK3 (green) and DNA (red) in oocytes at GV, GVBD, pro-M I, M I, A I and M II stages. (C) Oocytes at the metaphase I stage were incubated in M2 medium containing 10 µM taxol for 45 minutes and then double stained with antibodies against ERK3 as well as α-tubulin. Green, α-tubulin; red, ERK3; blue, DNA; yellow, overlapping of green and red. Each sample was counterstained with Hoechst 33258 to visualize DNA. Bar = 10 µm.

To investigate the role of ERK3 in mouse oocyte maturation, we first examined the dynamic distribution and expression of ERK3 at different stages. Western blots showed that ERK3 detected as the dark band was expressed at all stages of oocyte maturation (Fig. 1A). For the subcellular localization of ERK3, oocytes were processed for immunofluorescent staining at different stages of meiosis. ERK3 mainly distributed in the germinal vesicle at the GV stage. Shortly after GVBD (1–2 h of culture), ERK3 began to migrate to the periphery of chromosomes until the MI spindle was formed. At pre-MI, MI, ATI and MII stages, ERK3 stably localized to the spindle (Fig. 1B).

After observing that ERK3 mainly localized at the spindle after pre-MI,we investigated the correlation between ERK3 and microtubule dynamics. We used taxol, a microtubule-stabilizing agent, to treat oocytes. As shown in Figure 1C, the microtubule fibers in taxol-treated oocytes were excessively polymerized, leading to significantly enlarged spindles and numerous asters in the cytoplasm. In our experiments, ERK3 was detected on the fibers of the abnormal spindles as well as cytoplasmic asters.

### ERK3 deletion caused MI arrest during mouse oocyte maturation

**Figure 2:**
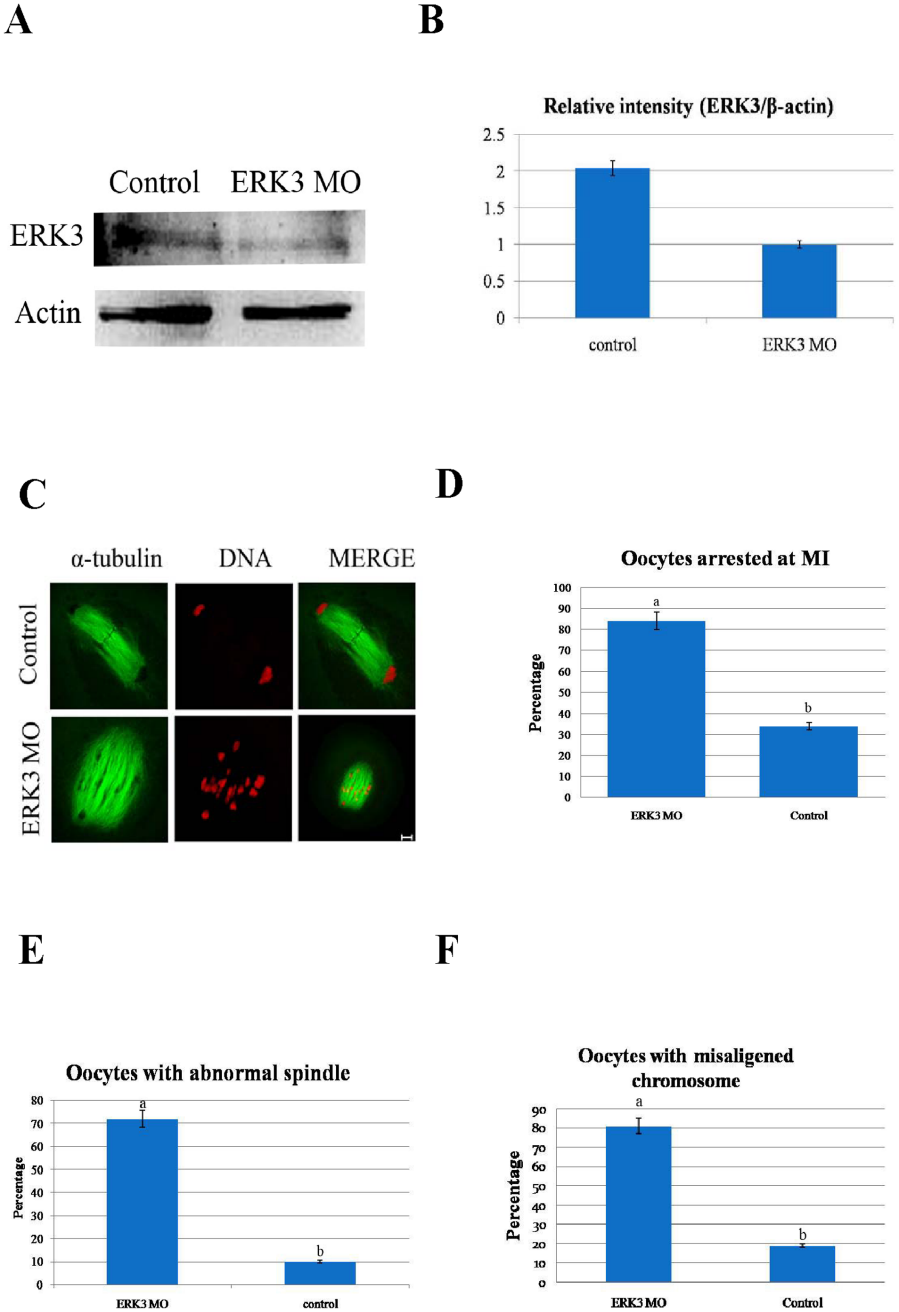
ERK3 deletion arrested oocytes at the MI stage and led to decreased spindle stability. After microinjection of ERK3 MO, the oocytes were incubated in M2 medium containing 2.5 µM milrinone for 21 h, then transferred to milrinone-free M2 medium for 10 h. (A) Western blot of ERK3 in the ERK3 MO group and control group. The ERK3 molecular mass is 100 kDa and that of actin is 42 kDa. (B) Relative intensity of ERK3/β-actin was assessed by volume analysis. (C) After microinjection, oocytes microinjected with ERK3 morpholino were arrested at the MI stage at 10 h of culture, but the control oocytes were in the AI stage. Double staining of α-tubulin (green) and DNA (red). Bar = 10 µm. (D) Percentage of oocytes in the ERK3 MO microinjected group (n = 42) and control group (n = 40). Data are presented as mean ± SE. Different superscripts indicate statistical difference (p<0.05). (E) Percentage of oocytes with abnormal spindles in the ERK3 MO injected group (n = 45) and control group (n = 44). (F) Percentage of oocytes with misaligened chromosomes in the ERK3 MO injected group (n = 45) and control morpholino injected group (n = 44). Data are presented as mean ± SE. Different superscripts indicate statistical difference (p<0.05).

To assess its function, ERK3 was knocked down by microinjection of ERK3 MO. Western blot and volume analysis revealed efficient deletion of ERK3 protein (Fig. 2A, B). Compared to oocytes microinjected with control morpholino (control), the expression of ERK3 was significantly reduced in oocytes microinjected with ERK3 MO (Fig. 2A,B). ERK3-deleted and control oocytes were cultured for 10 h, then stained with α-tubulin and PI to assess oocyte stages. Immunofluorescence and statistical analysis showed that 84% (84/100) oocytes in the ERK3 MO group were arrested at metaphase, while 66% (79/120) oocytes in the control group progressed to anaphase (Fig. 2C, D). Additionally,72% (72/100) of oocytes in the ERK3 MO group displayed abnormal spindles, but only 10% (12/120) of oocytes in the control group showed similar phenotypes (Fig. 2E). Moreover, the percentage of oocytes with misaligned chromosomes in ERK3 MO and control groups were 81% (81/100) and 19% (23/120), respectively (Fig. 2F). Different superscripts indicate statistical difference (p<0.05).

### ERK3 deletion disrupted the attachment between microtubules and chromosomes

**Figure 3:**
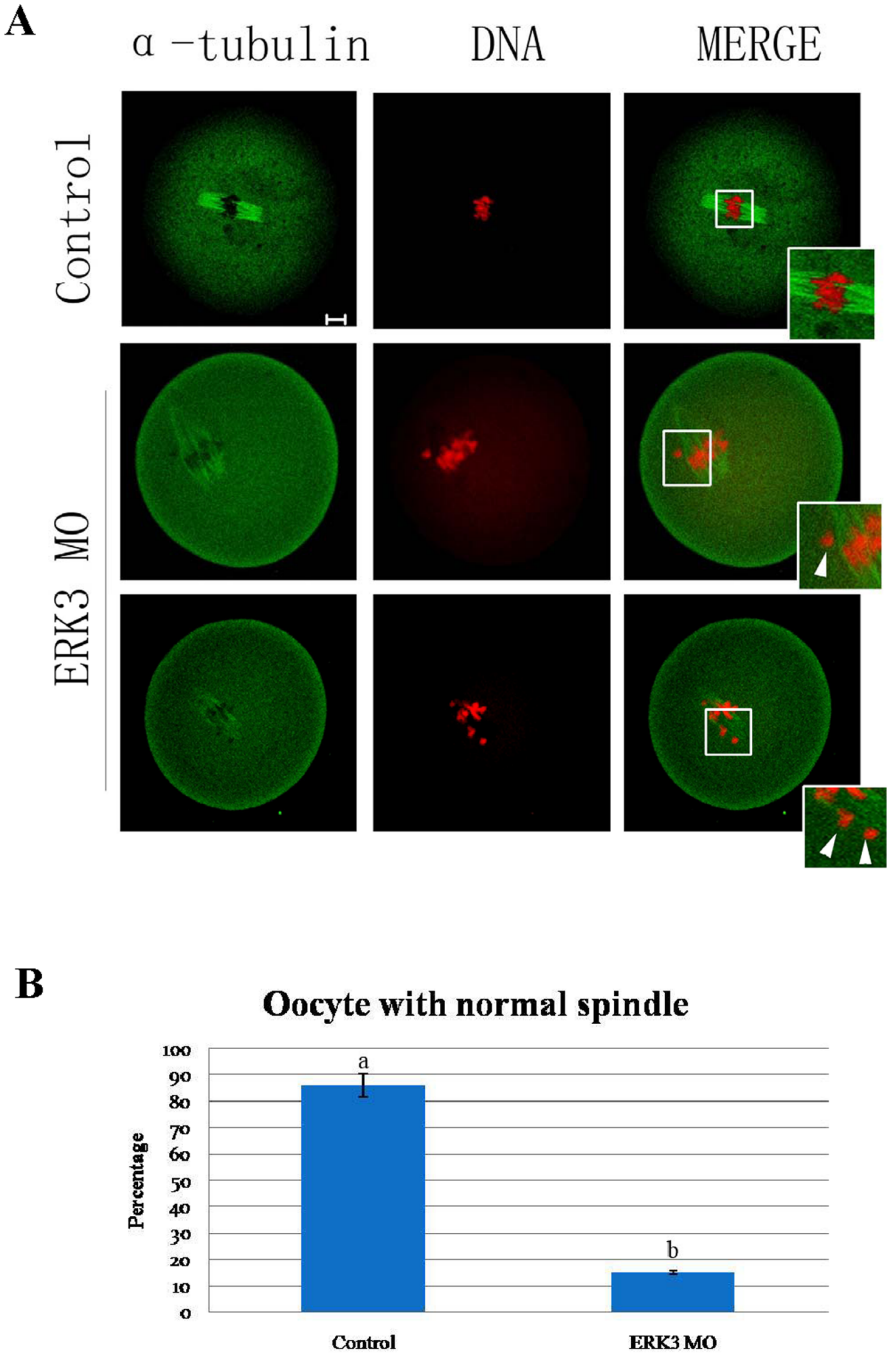
Deletion of ERK3 induced unstable microtubule-chromosome attachments at the MI stage. (A) Oocytes of control and ERK3 MO groups were cultured for 8.5 h followed by cold treatment for 10 minutes in M2 medium which was pre-cooled at 4°C. Magnifications of the boxed regions show that all chromosomes were attached to microtubules in control oocytes, but not in ERK3 MO microinjected oocytes, whereas abnormal spindles were observed in ERK3 MO group. Bar = 10 µm. (B) Percentage of oocytes with normal spindles in the ERK3 MO group (n = 50) and control group (n = 49). Data are presented as mean ± SE. Different superscripts indicate statistical difference (p<0.05). Bar = 10 µm.

The results above showed that ERK3 might be involved in the regulation of the metaphase –anaphase transition, so we used low temperature treatment to explore details. ERK3-deleted and control oocytes were cultured for 8.5 h, and then transferred to M2 medium which was pre-cooled to 4°C and cultured for 10 minutes. As shown in Figure 3A, all chromosomes were attached to microtubules in control oocytes; interestingly, chromosomes in ERK3-deleted oocytes were disordered; magnification of the boxed region shows that some chromosomes were not attached to microtubules. Notably, 86% (45/52) oocytes in the control group displayed normal spindles, but only 15% (9/60) oocytes in the ERK3 MO group had normal spindles because of the disruption of microtubule-chromosome attachments (Fig.3B). Different superscripts indicate statistical difference (p<0.05).

**Figure 4:**
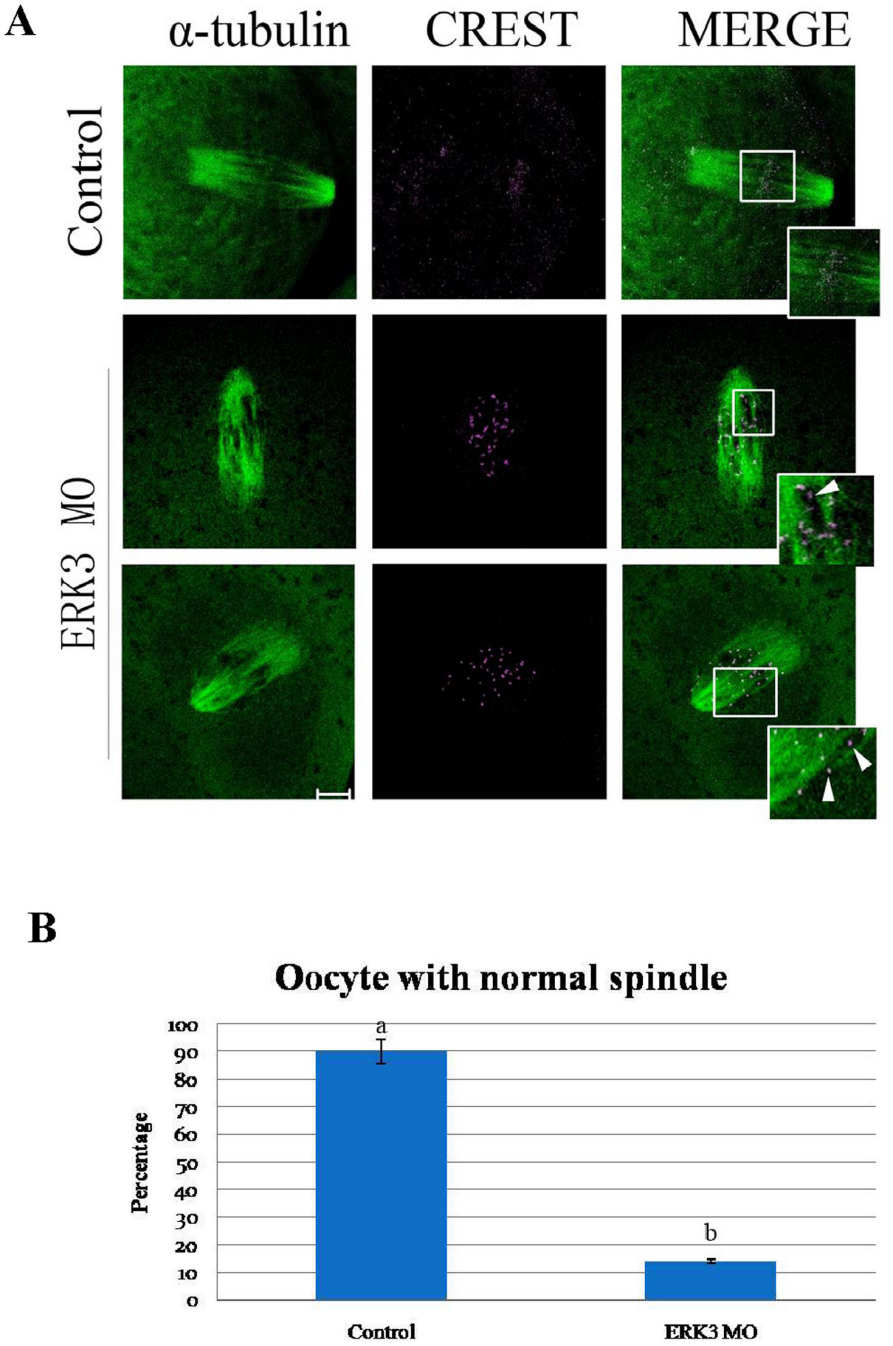
ERK3 deletion disrupted the attachments between kinetochores and microtubules. (A) Oocytes of control and ERK3 MO groups were cultured for 8.5 h followed by cold treatment for 10 minutes in M2 medium which was pre-cooled at 4°C. Magnifications of the boxed regions showed intact attachments between kinetochores and microtubules in the control group, but not in the ERK3 MO group, whereas abnormal spindles were observed in the ERK3 MO group. Bar = 10 µm. (B) Percentage of oocytes with normal spindles in the control group (52) and the ERK3 MO group (51). Data are presented as mean ± SE. Different superscripts indicate statistical difference (p<0.05).

We further proved that ERK3 deletion disrupted the attachments between microtubules and chromosomes. Carrying out the same low temperature treatment, the oocytes were stained with CREST (anticentromere antibody, autoimmune sera from patients with calcinosis, Raynaud phenomenon, esophageal dysmotility, sclerodactyly, and telangiectasia, a marker of CENPs at the kinetochores) and α-tubulin. The results verified that the attachments were destroyed, some spindle fibers were scattered in the cytoplasm, some kinetochores appeared to be disordered; the magnified box region shows details of disruption (Fig.4A). Only 14% (6/42) oocytes displayed normal spindles in the ERK3 MO group, compared to 90% (45/50) in the control group (Fig. 4B). Different superscripts indicate statistical difference (p<0.05).

### ERK3 deletion prevented chromosome segregation and activated the SAC protein

**Figure 5:**
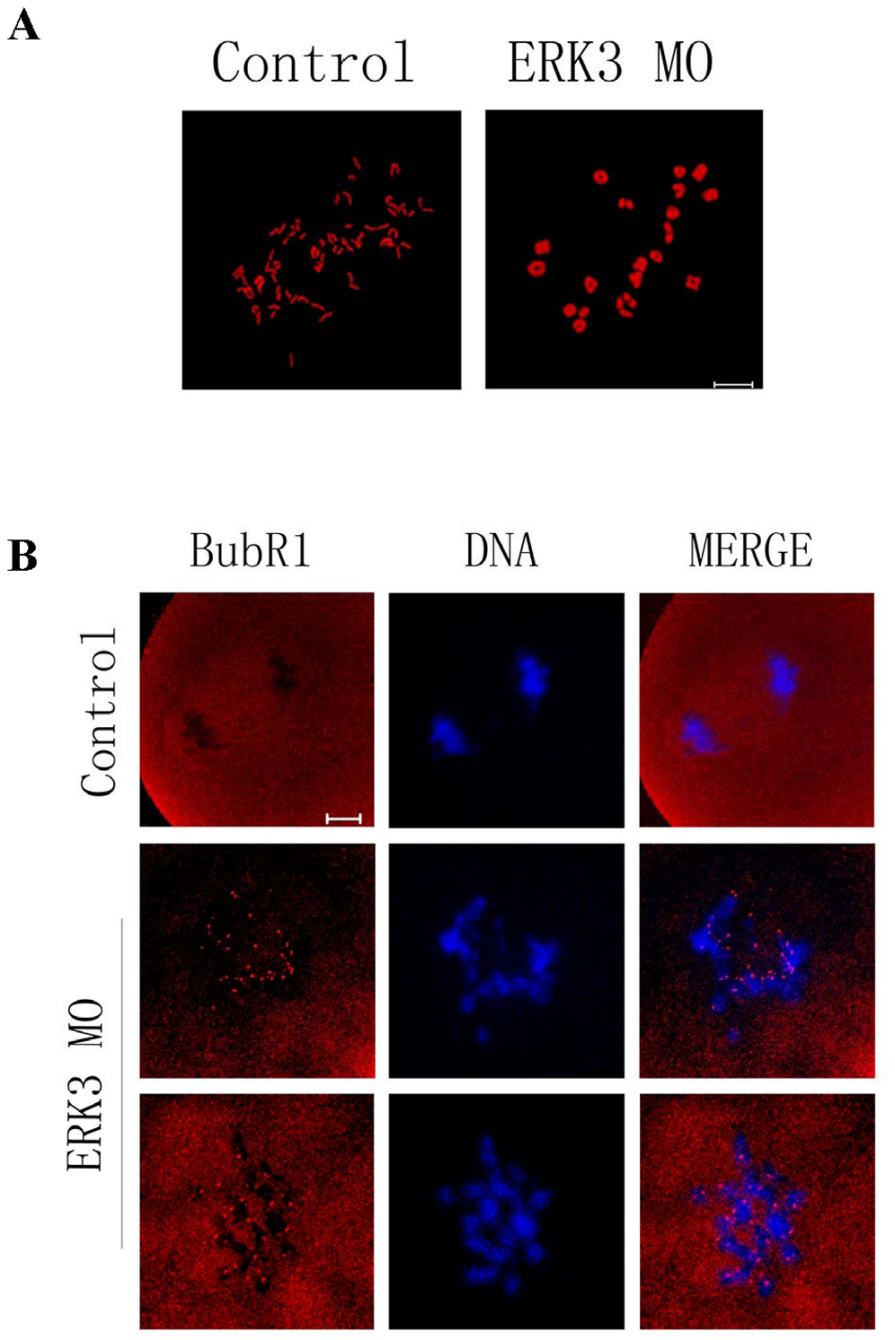
ERK3 deletion inhibited chromosome segregation and activated SAC protein BubR1. (A) Oocytes of control and ERK3 MO groups were cultured for 10 h, followed by chromosome spreading experiments. (B) Detection of BubR1 in oocytes in control and ERK3 MO groups. Red, BubR1; blue, DNA. Bar = 10 µm.

Since ERK3 deletion disrupted the microtubule-kinetochore attachments, we asked whether the chromosomes could undergo correct segregation. Oocytes in both ERK3 MO and control groups were cultured for 10 h. Chromosome spreading showed that all chromosomes were still in the bivalent state in ERK3 deleted oocytes(14/14), while univalent chromosomes could be seen in control oocytes(13/15), indicating completion of anaphase (Fig. 5A). Stable microtubule-kinetochore attachment is critical for the correct chromosome segregation, so the failure of chromosome segregation in ERK3 deleted oocytes might be caused by the loss of protection of ERK3 for K-MT attachment. Since a lack of stable interactions between K-MTs was observed in the above experiments, we assessed the localization of BubR1 in oocytes from the ERK3-deleted group. Specific signals for BubR1 were detected in the MI arrest oocytes in the ERK3 MO group, while the control group showed no signals for BubR1. Detection of BubR1 indicates spindle assembly checkpoint activation.

## Discussion

In this study, we for the first time demonstrate that ERK3 is important for MI spindle stability and required for the metaphase-anaphase transition in mouse oocyte maturation. Deletion of ERK3 in oocytes using specific morpholino disrupted MI spindle organization, and caused MI arrest.

Currently, most studies on ERK3 focus on its functions in mitosis, and ERK3-deficient mice have been found to have neonatal lethality[20]; therefore, we want to dig up the function of ERK3 in meiosis, especially during mouse oocyte meiotic maturation. ERK1/2 are co-expressed in all mammalian tissues and implicated as key regulators of cell proliferation and differentiation as well as oocyte maturation in culture[25], [26]; ERK3 and ERK1/2 belong to the MAP kinase family. We proposed that ERK3 might participate in cell cycle regulation. First we labeled ERK3 with antibody to study the expression and localization of ERK during mouse oocyte maturation. Immunofluorecent analysis showed that ERK3 localized to the spindle, which indicates that ERK3 might function in microtubule organization and spindle assembly. Taxol treatment polymerized microtubule fibers and led to significantly enlarged spindles, together with numerous asters in the cytoplasm; ERK3 signals were detected to co-localize with α-tubulin of the spindle and asters (Fig. 1C). To further explore the function of ERK3, we used specific morpholino to delete ERK3 expression. High incidence of abnormal spindles was observed in ERK3 deleted oocytes. We conclude that ERK3 is crucial for meiotic spindle assembly.

Accurate chromosome separation ensures proper distribution of genetic material during cell division in mitosis and meiosis[27]. Mammalian oocytes are not able to progress through the MI stage until all chromosomes have been properly attached to the bipolar spindle and are aligned at the metaphase plate[28]. SAC proteins including BubR1 and Bub3 ensure correct segregation of homologous chromosomes and provoke a cell cycle arrest in metaphase if any chromosome is not correctly attached to the bipolar spindle[29], [30], [31]. After nuclear envelope breakdown in animal cells, highly dynamic centrosome-nucleated microtubules continuously probe the cytoplasm with their plus ends to search and capture chromosomes[32], [33], [34]. Microtubules that encounter a kinetochore become stabilized, whereas those that do not soon depolymerize[35]. In this case, we found that ERK3 deletion arrested oocyte meiosis at the MI stage (Fig.2C), and misaligned chromosomes were also observed. We then used low temperature treatment to further explore the correlation between microtubules and kinetochores; the results showed that ERK3 deletion disrupted the attachments between microtubules and kinetochores labeled with CREST and chromosomes (Fig.4B). To prevent aneuploidy, the cell generates a ‘wait-anaphase’ signal known as the SAC that inhibits anaphase onset until all kinetochores achieve biorientation, and tension is established between sister kinetochores[9]. BubR1 was detected in ERK3 deleted oocytes, even though the oocytes had been cultured for 10 h. Activation of SAC proteins resulted in MI arrest (Fig.5B). This result showed that damage of the attachments between microtubules and kinetochores caused by ERK3 activated SAC followed by metaphase arrest. We propose that ERK3 deletion causes disruption of K-MT attachments, therefore activation of SAC, and finally metaphase arrest. Studies in several model systems have proposed that the metaphase anaphase transition is induced by APC/C and the downstream target of the spindle checkpoint is Cdc20, which is an 11-subunit complex containing ubiquitin ligase activity[36]. The mitotic spindle checkpoint transmits inhibitory signals to APC/C^Cdc20^, stabilizing securin (Pds1) and cyclin B, and thus prevents the metaphase-anaphase transition until all chromosomes have established a bipolar attachment to the spindle[37]. In oocyte meiosis, Cdc 20 was proved to be essential for the transition from MI to AI [38]. It has been shown that phosphatases Cdc14A and Cdc14B binds to ERK3 and reverse its C-terminal phosphorylation in mitosis and Cdc14A regulates oocyte maturation[39], The relationship between ERK3 and Cdc20 or between ERK3 and Cdc14A could be the next step for ERK3 function in oocyte meiosis.

All data show that ERK3 is essential for spindle stability and required for metaphase-anaphase transition in mouse oocyte meiosis.

## Materials and Methods

All chemicals and culture media were purchased from Sigma Chemical Company (St. Louis, MO) except for those especially mentioned.

### Ethics Statement

4–6 week-old KM mice care and use were conducted in accordance with the Animal Research Committee guidelines of the Institute of Zoology, Chinese Academy of Sciences. The institute does not issue a number to any animal study, but there is an ethical committee to guide animal use. Each study requires the permit to use animals from the committee. The animal facility must get licensing from the experimental animal committee of Beijing city. The animal handling staff (including each post-doc and doctor student) must be trained before using animals. Mice were housed in a temperature-controlled room with proper darkness-light cycles, fed with a regular diet, and maintained under the care of the Laboratory Animal Unit, Institute of Zoology, Chinese Academy of Sciences. The mice were killed by cervical dislocation. The only procedure performed on the dead animals is the collection of oocyte from the ovary.

### Oocyte collection and culture

The only procedure performed on the dead animals is the collection of oocyte from the ovary. Oocytes were collected in M2 medium supplemented with 2.5 µM milrinone to maintain them at the germinal vesicle (GV) stage. Then oocytes were washed 6 times to wash out the effect of milrinone and cultured in M2 medium to GV, GVBD, Pro-MI, MI, ATI, MII stages.

### Taxol and cold treatment of oocytes

For taxol treatment, 5 mM taxol in DMSO stock was diluted in M2 medium to achieve a final concentration of 10 µM, and oocytes were incubated for 45 min. After treatment, oocytes were washed thoroughly and used for immunofluorescent experiments. Control oocytes were treated with the same concentration of DMSO in the medium before examination.

### Immunofluorescence and confocal microscopy

Oocytes were fixed with 4% paraformaldehyde/PBS (pH 7.4) for at least 30 min. After being permeabilized with 0.5% Triton X-100 at room temperature for 20 min, oocytes were blocked in 1% BSA-supplemented PBS for 1 h and then incubated with rabbit anti-ERK3 antibody (Santa Cruz; 1∶100), human anti-CREST antibody (Fitzgerald; 1∶50), or anti-α-tubulin antibody (Sigma; 1∶200), respectively, overnight at 4°C. After three washes with PBS containing 0.1% Tween 20 and 0.01% Triton X-100 for 5 min each, the oocytes were labeled with FITC conjugated goat-anti-rabbit IgG (Zhong Shan Jin Qiao; 1∶100), TRITC conjugated goat-anti-rabbit IgG (Zhong Shan Jin Qiao; 1∶100), Cy5-anti-human IgG (Jackson; 1∶100) or FITC-anti-mouse IgG (Zhong Shan Jin Qiao; 1∶100) for 1 h at room temperature and then washed three times with PBS containing 0.1% Tween-20 and 0.01% Triton X-100. The oocytes were co-stained with Hoechst 33342 or PI. Finally, the oocytes were mounted on glass slides and examined with a confocal laser scanning microscope (Zeiss LSM 510 META, Germany).

### Microinjection of ERK3/control morpholino antisense oligos

Microinjections were performed using a Nikon Diaphot ECLIPSE TE 300 (Nikon UK Ltd., Kingston upon Thames, Surrey, UK) and completed within 30 minutes. 1 mM ERK3 MO antisense oligos (GENE TOOLS, LLC, CGAATTTCTCTGCCATTTTGAAACC) were microinjected into the cytoplasm to delete ERK3. The same amount of negative control morpholino (GENE TOOLS, LLC) was also injected as control. After microinjection, the oocytes were arrested at the GV stage for 21 h in M2-containing 2.5 µM milrinone to knock down ERK3. Each experiment consisted of three separate replicates and approximately 300 oocytes were injected in each group.

### Chromosome spreading

For chromosome spreading, oocytes were left for 10 minutes in 1% sodium citrate at room temperature and then fixed with fresh methanol: glacial acetic acid (3∶1), 10 mg/ml PI was used for chromosome staining. Cells were examined with a Confocal Laser Scanning Microscope. Instrument settings were kept constant for each replicate.

### Immunoblotting analysis

Immunoblotting was performed as described previously[40]. Briefly, 300 mouse oocytes were collected in SDS sample buffer and heated for 5 min at 100°C. The proteins were separated by SDS-PAGE and then electrically transferred to polyvinylidene fluoride membranes. Following transfer, the membranes were blocked in TBST containing 5% skimmed milk for 2 h, followed by incubation overnight at 4°C with rabbit polyclonal anti-ERK3 antibody (1∶500) and mouse monoclonal anti-β-actin antibody (1∶1000). After washing three times in TBST, 10 minutes each, the membranes were incubated for 1 h at 37°C with peroxidase-conjugated rabbit anti-rabbit IgG (1∶1000) and peroxidase-conjugated rabbit anti-mouse IgG, respectively. Finally, the membranes were processed using the SuperSignal West Femicrotubuleo maximum sensitivity substrate (Thermo Scientific).

### Statistical analysis

Data (mean ± SE) were from at least three replicates per experiment and analyzed by ANOVA using SPSS software (SPSS Inc, Chicago, IL) followed by Student-Newman-Keuls test. Difference at P<0.05 was considered to be statistically significant and different superscripts indicate the statistical difference.
